# Multiple Routes and Milestones in the Folding of HIV–1 Protease Monomer

**DOI:** 10.1371/journal.pone.0013208

**Published:** 2010-10-13

**Authors:** Massimiliano Bonomi, Alessandro Barducci, Francesco L. Gervasio, Michele Parrinello

**Affiliations:** 1 Computational Science, Department of Chemistry and Applied Biosciences, ETH Zurich, Lugano, Switzerland; 2 Computational Biophysics Group, Structural Biology and Biocomputing Programme, Spanish National Research Centre, Madrid, Spain; Massachusetts Institute of Technology, United States of America

## Abstract

Proteins fold on a time scale incompatible with a mechanism of random search in conformational space thus indicating that somehow they are guided to the native state through a funneled energetic landscape. At the same time the heterogeneous kinetics suggests the existence of several different folding routes. Here we propose a scenario for the folding mechanism of the monomer of HIV–1 protease in which multiple pathways and milestone events coexist. A variety of computational approaches supports this picture. These include very long all-atom molecular dynamics simulations in explicit solvent, an analysis of the network of clusters found in multiple high-temperature unfolding simulations and a complete characterization of free-energy surfaces carried out using a structure-based potential at atomistic resolution and a combination of metadynamics and parallel tempering. Our results confirm that the monomer in solution is stable toward unfolding and show that at least two unfolding pathways exist. In our scenario, the formation of a hydrophobic core is a milestone in the folding process which must occur along all the routes that lead this protein towards its native state. Furthermore, the ensemble of folding pathways proposed here substantiates a rational drug design strategy based on inhibiting the folding of HIV–1 protease.

## Introduction

**Figure 1:**
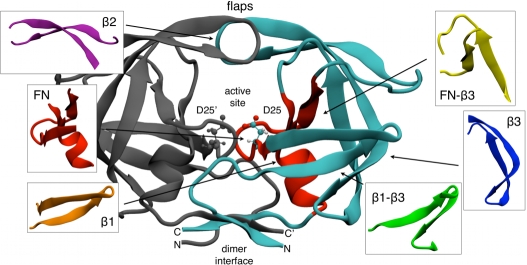
Structure of the HIV–1–PR dimer (PDB code 1BVG). The active site is situated at the interface between the two identical subunits of the homodimer. It is characterized by the sequence Asp-Thr-Gly (Asp25, Thr26 and Gly27), common to aspartic proteases. The two Asp25 residues act as the catalytic residues. At the top, in evidence the flexible flaps region. In the small boxes, the six main motifs in which we classified the native contact map in Fig. 2.

The protease of Human Immunodeficiency Virus type 1 (HIV–1–PR) is a dimer in its catalytic competent form (Fig. 1). Each of the two identical monomers has a single domain composed of 99 amino acids. Several experimental [1]–[3] and computational studies [4], [5] suggest that the folding of this enzyme is a three-state process in which first two monomers fold independently and then dock in the dimer native state. Studying the folding of the HIV–1–PR monomer is therefore the first step in the comprehension of the whole enzyme formation.

Understanding protein folding at an atomic resolution is a fundamental yet challenging task for both experiments and simulations. In the case of HIV–1–PR, characterizing the ensemble of folding pathways would also provide precious information for the rational design of novel anti-HIV drugs. HIV–1–PR is one of the main targets of Acquired Immuno-Deficiency Syndrome therapies as it performs an essential function in the HIV life cycle by cleaving the viral poly-protein and producing the components that are needed for the mature virus assembly. The virus very high mutation rate is capable of eluding the effects of competitive inhibition based drugs in a very short time [6]. An alternative strategy for neutralizing the HIV–1–PR function consists in inhibiting the formation of the protease by interfering either with the folding process of the monomer or with its dimerization [7], [8]. Several lines of evidence show that the unfolded protein, the monomer and the dimer although separated by large barriers have comparable energies [3]. Furthermore, it has been suggested that the dimer is stabilized by the substrate. In such a scenario it would be more advantageous to target the monomer folding.

In light of these considerations, a deep understanding of the monomer folding mechanism would have broader significance. To this end many theoretical and experimental studies have been performed [9]–[11]. Among the first group, a possible scenario has been suggested by off-lattice G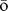 models simulations in which first the fragments 24–34 (S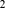) and 83–93 (S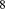) fold to form the so-called Local Elementary Structures (LES) and subsequently these LES dock in the folding nucleus (FN) [12], [13]. Recently we have shown by fully atomistic molecular dynamics (MD) and metadynamics [14] simulations the stability of the LES in solution and calculated the strength of their interaction, adding further evidence to the their central role in monomer folding [15]. It has also been proposed that a peptide mimicking the sequence of one of the two LES (p–S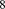) could be used as efficient folding inhibitor [12]. Remarkably, these theoretical predictions have been confirmed by *in*–*vitro* and *ex*–*vivo* experiments [16], [17]. Standard enzymatic assays indicate that p–S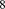 inhibits the HIV–1–PR with an inhibition constant K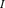 = 2.58
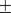
0.78
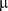
M [16], while results on infected cells indicate that this peptide is not cytotoxic and inhibits the maturation of the virus at a micromolar concentration [17]. These successes further increase the need for a thorough investigation of the folding intermediates and of all the possible folding pathways.

Here we use state-of-the-art computational techniques to formulate a plausible scenario for the folding mechanism of HIV–1–PR monomer. First we performed 0.5 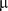
s long all-atom MD simulations in explicit solvent at room temperature. Then we run multiple high-temperature unfolding simulations, clustering the structures visited and analyzing the network of transitions between clusters. Finally, we used a recently developed structure-based potential at atomistic resolution [18] together with a combined parallel tempering [19] and metadynamics [20] (PTMetaD) [21] to obtain well converged multidimensional free-energy surfaces (FES). Especially effective has proven the application of the very recently developed well-tempered ensemble (WTE) [22].

The all-atom simulations and structure-based potential FES revealed a complex scenario respectively for the high-temperature unfolding and folding mechanism of this protein. Both processes were characterized by the simultaneous presence of multiple pathways and milestone events. In either cases, heterogeneity could be ascribed to the behavior of particular 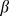-hairpin subunits, while the milestone events corresponded to the disruption/formation of an extended folding nucleus composed by the two LES S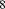 and S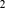 plus another hydrophobic fragment (residues 73–80). This remarkable agreement between the nature and the fine details of the high-temperature unfolding process and folding mechanism prompted us to formulate valuable hypothesis about the actual folding routes of the HIV–1 monomer.

These results obtained here for the HIV–1–PR monomer might have a more general valence. In fact one finds that folding is guided by a milestone event which occurs rather rapidly reducing the conformational space that needs to be sampled. On the other end, the heterogeneous nature of the overall process is consistent with a body of experimental evidence on single domain protein folding.

## Results

**Figure 2:**
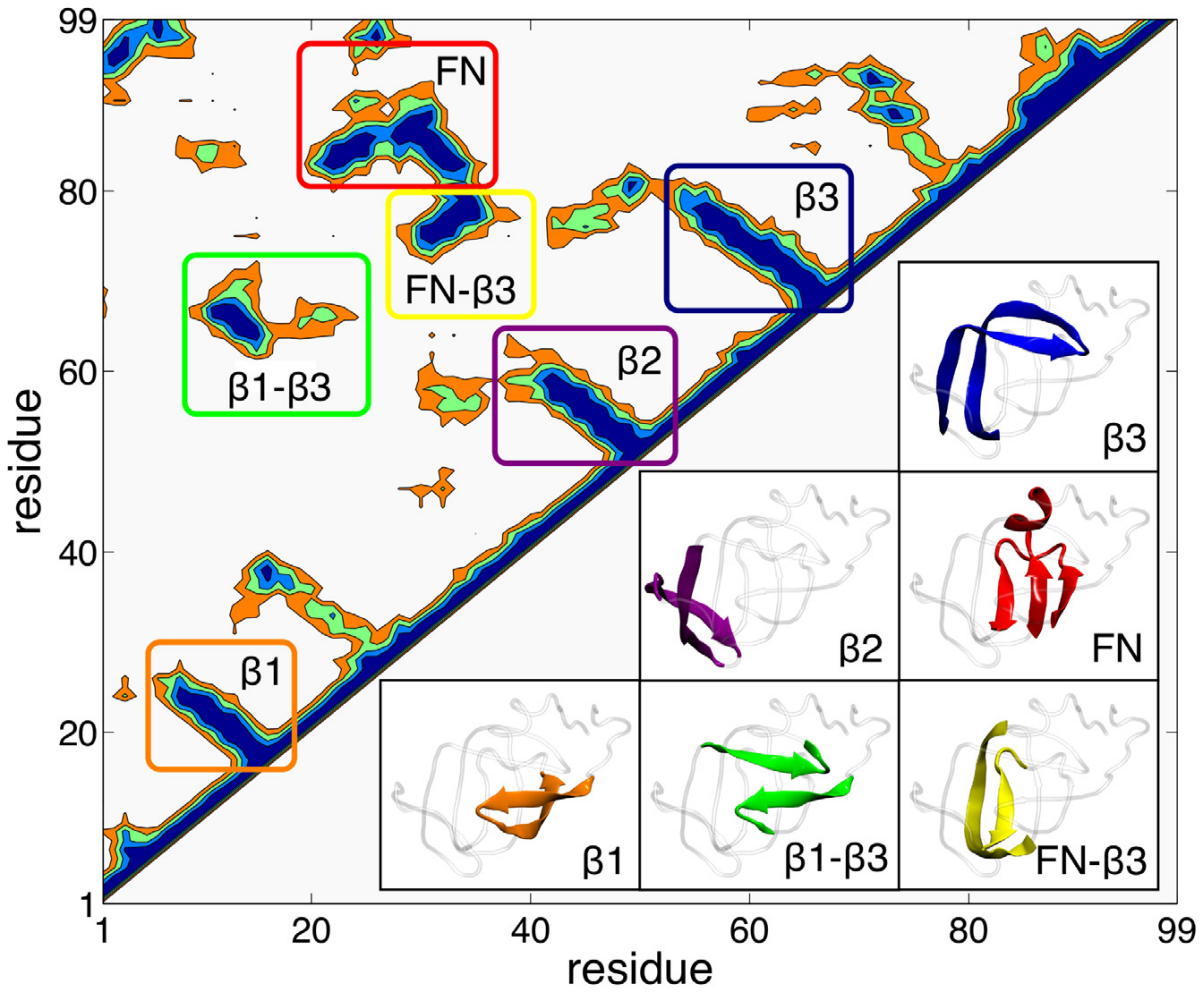
Contact map of HIV–1–PR in its native state. We classified the contacts in six groups: in orange 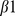 (residues 10–23), in violet 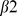 (residues 41–58), in blue 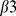 (residues 55–75), in red FN (residues 24–34 and 83–92), in green 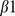
–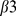 and in yellow FN–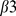.

While models of different complexity were used, a common description of the protein in terms of native contacts was adopted throughout all this study (see *Methods* section). In Fig. 2 we show the contact map of HIV–1–PR in its native state. After extensive analysis of both structure and dynamics, we classified these contacts into six main groups. The three 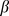
–hairpin structures are labeled as 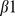 (residues 10–23), 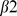 (residues 41–58) and 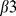 (residues 55–75). We also label different set of interactions, namely 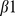
–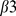 stands for the interaction between 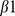 and 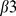, while FN refers to the interaction between fragments 24–34 and 83–92. This group corresponds to the folding nucleus [12], [15]. Finally the interaction FN–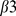 will be referred to below. The fragment 83–93, which is a very stable 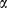-helix [15], was not studied as a separate entity but in relation to its role in the FN structure.

In the following sections we analyze separately the results of our all-atom and coarse-grained simulations. In the *Discussion*, on the basis on these results we propose a plausible scenario for the folding mechanism of the monomer.

### All-atom simulations

Starting from the crystallographic structure, we performed a 512 ns long NVT simulation of HIV–1–PR monomer at 300K using an all-atom representation for the protein and for the solvent degrees of freedom. The simulation was performed to confirm the stability of the native state of the protein and study the relative fluctuations of the various motifs of the fold. Details of the simulation protocol are reported in the *Methods* section. The structure of the monomer was stable during the whole NVT simulation. The root mean square fluctuations of the C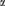 atoms were within 0.5 and 1.5 Å along most of the chain (Figure S1). Three regions displayed a greater mobility: the C and N termini and the 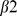 fragment.

The terminal regions displayed fluctuations of the order of 3 to 4 Å. These values are much smaller than those reported in Ref. [5]. There the authors found values ranging from 5 to 10 Å in a much shorter (5 ns) simulation. The difference can be explained either by the different force field used, AMBER99SB here and CHARMM in Ref. [5], or by the longer time-scale explored by our simulation. We incline to the second hypothesis. After several tens of nanoseconds we observed a partial assembly of the N and C termini into a 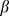
–sheet structure (Figure S2). This rearrangement most likely made the structure more rigid. What is more, the formation of such 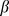 structure was also observed in a longer simulation that used the GROMOS force field [23], adding weight to our suggestion.

The flexibility of 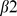 is not surprising. This motif corresponds in the dimer structure to one of the two flaps (Fig. 1), a region that has been shown both experimentally [24], [25] and computationally [26]–[28] to be extremely flexible. This flexibility is indeed functional to the enzymatic activity. The structures we focused on remained stable during the simulation with the exception of 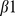
–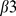 which underwent a fluctuation in which this interaction was broken and reformed in about 10 ns (Figure S2 and Figure S3). The most stable behavior was instead shown by 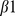, which exhibited the smallest fluctuations.

We then performed multiple high-temperature unfolding simulations starting from the native conformation. It has been suggested that high-temperature unfolding MD simulations can be used to formulate useful hypothesis for experimental studies on folding [29]–[31].

The configurations visited in the unfolding simulations were clusterized on the basis of the number of native contacts formed using the k-means algorithm [32]. Details of the unfolding simulations, the clustering algorithm and the network analysis can be found in the *Methods* section.

**Figure 3:**
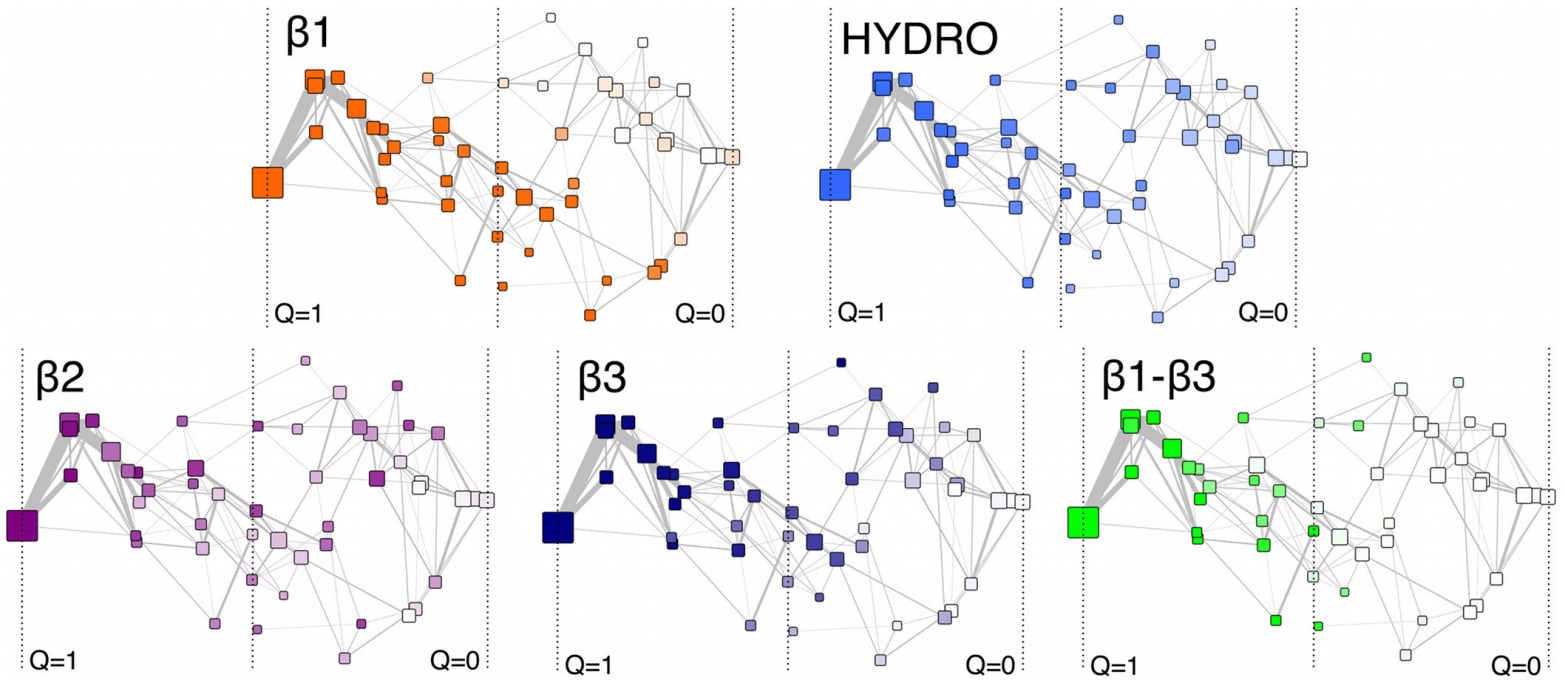
Network of clusters from all-atom high-temperature unfolding trajectories. Clusters are positioned according to the metric multidimensional scaling algorithm [59]. The size of the area of each cluster is proportional to its population, the link size to the number of trajectories connecting two clusters. 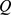 is the total number of contacts. From left (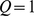) to right (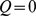), clusters contain conformations more and more unfolded. The vertical dotted lines are drawn at 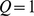, 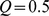 and 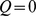 as a guide for the eye. In each panel, clusters are colored with an intensity which is (linearly) proportional to the number of contacts that belong to a certain motif and that are still formed at that particular stage of the overall unfolding of the protein. The set HYDRO is composed by the group of contacts FN and FN–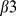, which were grouped together since they displayed a similar behavior along the unfolding pathways.

The analysis of the clusters network reveals the existence of at least two distinct unfolding pathways (Fig. 3). The main difference between the two routes is determined by 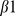 (Fig. 3, panel 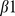). In the most populated pathway, this hairpin is the last secondary structure to unfold, while in the other pathway it unfolds in the early stages. The folding of 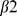 (Fig. 3, panel 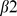) is uncorrelated to the overall unfolding of the protein. Folded and unfolded 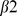 configurations can be found at different stages along the two main pathways. The hairpin 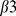 (Fig. 3, panel 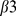) unfolds later than the other motifs in both the dominant pathways, only when almost 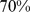 of the protein is unfolded. The interaction 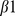
–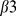 is quite weak and breaks at the early stages of unfolding (Fig. 3, panel 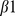
–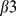). If we take this information together with that of panels 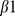 and 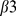, we can conclude that in the main pathway, when the contacts between the two hairpins are broken, each of the two 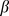-strands remains folded. In the alternative route, the inter-hairpin contacts are lost almost in sync with 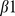 unfolding, while 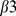 remains structured for longer times. The sets of contacts FN and FN–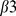, which involve a large number of hydrophobic residues buried inside the enzyme, are the last to unfold (Fig. 3, panel HYDRO).

### Structure-based potential simulations

The calculation of all-atom folding FES in explicit solvent is still impractical for this system, despite the increasing computational power [33] and the variety of enhanced sampling methods available [34], [35]. Not only for this reason but also to have an independent view on the folding mechanism, we chose to adopt a simplified potential energy function that is consistent with our representation of the protein in terms of native contacts. We used the structure-based potential recently introduced by Whitford *et al.* [18]. Despite its simplified nature, this potential has been shown to predict the folding mechanism of the B domain of Protein A, of the SH3 domain of C-Src Kinase and of Chymotrypsin Inhibitor 2, in agreement with other C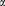 G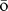 models and all-atom force fields. The success of this potential is probably connected to the fact that evolution has led proteins to display funneled energy landscapes with small degrees of ruggedness. This means that evolution has optimized the protein sequence in order to ensure a robust folding such that the native state does not have to compete with denatured conformations [36]–[40]. Thus a model based on the topology of the native state can be very effective in predicting the folding mechanism of proteins. What is more, a funneled landscape does not preclude the presence of multiple kinetically relevant folding routes, as it has been shown in various studies [41]–[44].

In the spirit of Ref. [45], in order to understand the folding mechanism we calculated multiple two-dimensional (2-D) FES as a function of the fraction of native contacts of our six motifs and at several temperatures across the model folding temperature (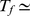
113.5K). To this effect we used PTMetaD boosted by WTE [22] and in combination with the reweighting technique of Ref. [46] (see *Methods* section).

Before analyzing the 2-D FES, let us discuss briefly the stability of the three 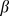-strand structures (Figure S6). Throughout the range of all temperatures, 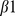 appears the most stable among the 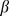-strands. For temperatures lower than 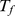, the FES of the hairpin 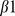 and 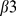 display bimodal distributions corresponding to folded and unfolded states that are sharper than 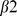. This suggests that, even at low temperature, 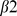 is more flexible than the other hairpin-like subunits of the monomer.

**Figure 4:**
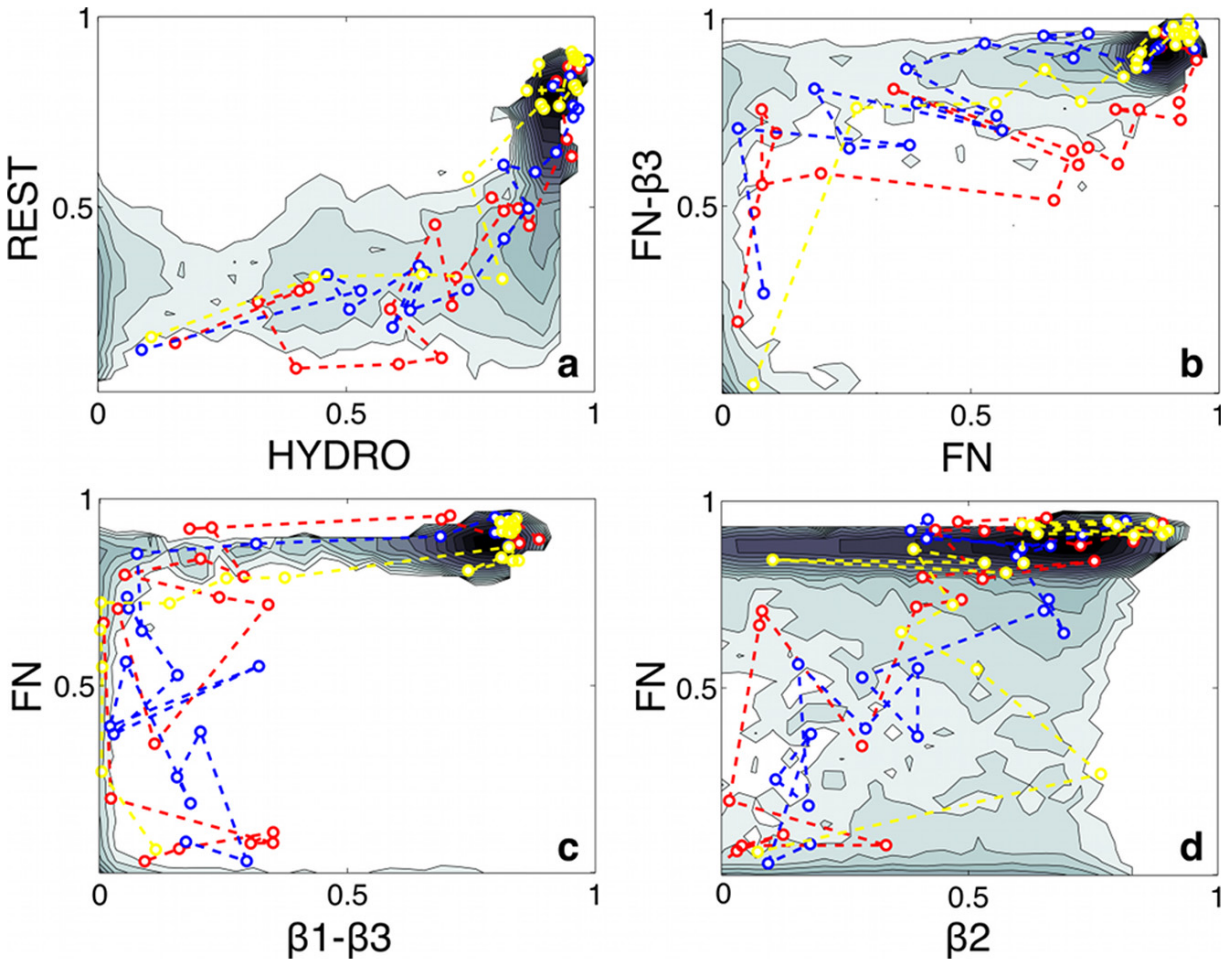
FES as a function of the fraction of native contacts of the six motifs at T = 0.969 obtained by reweighting the structure-based potential PTMetaD simulation. **a**. HYDRO vs. all the other contacts. **b**. FN vs. FN–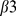. **c**. 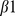
–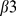 vs. FN. **d**. 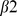 vs. FN. Isoenergy lines are drawn every 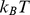. In dashed lines, projection of the relevant part of the all-atom explicit-solvent unfolding simulations at 700K (blue and red) and 500K (yellow).

The set of 2-D FES provides a clear explanation for the sequence of events that characterize the folding mechanism at the different temperatures. The hydrophobic core of the monomer composed by the set of contacts FN and FN–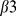 is the first structure formed during the folding process. In fact, if we analyze the FES as a function of the hydrophobic contacts and all the other variables (Fig. 4a), we see a clear L-shaped landscape. This indicates that first the hydrophobic collapse takes place and only after the rest of the structure is formed. The L-shape, which is very sharp at low temperature, becomes less definite as the temperature increases, but it is still clearly recognizable (Figure S7). This is a further proof that the hydrophobic collapse is a fundamental milestone in the folding process. If we analyze the single motifs contributing to the hydrophobic core, we notice that the contacts FN–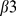 are formed before FN (Fig. 4b) independently of temperature (Figure S8).

**Figure 5:**
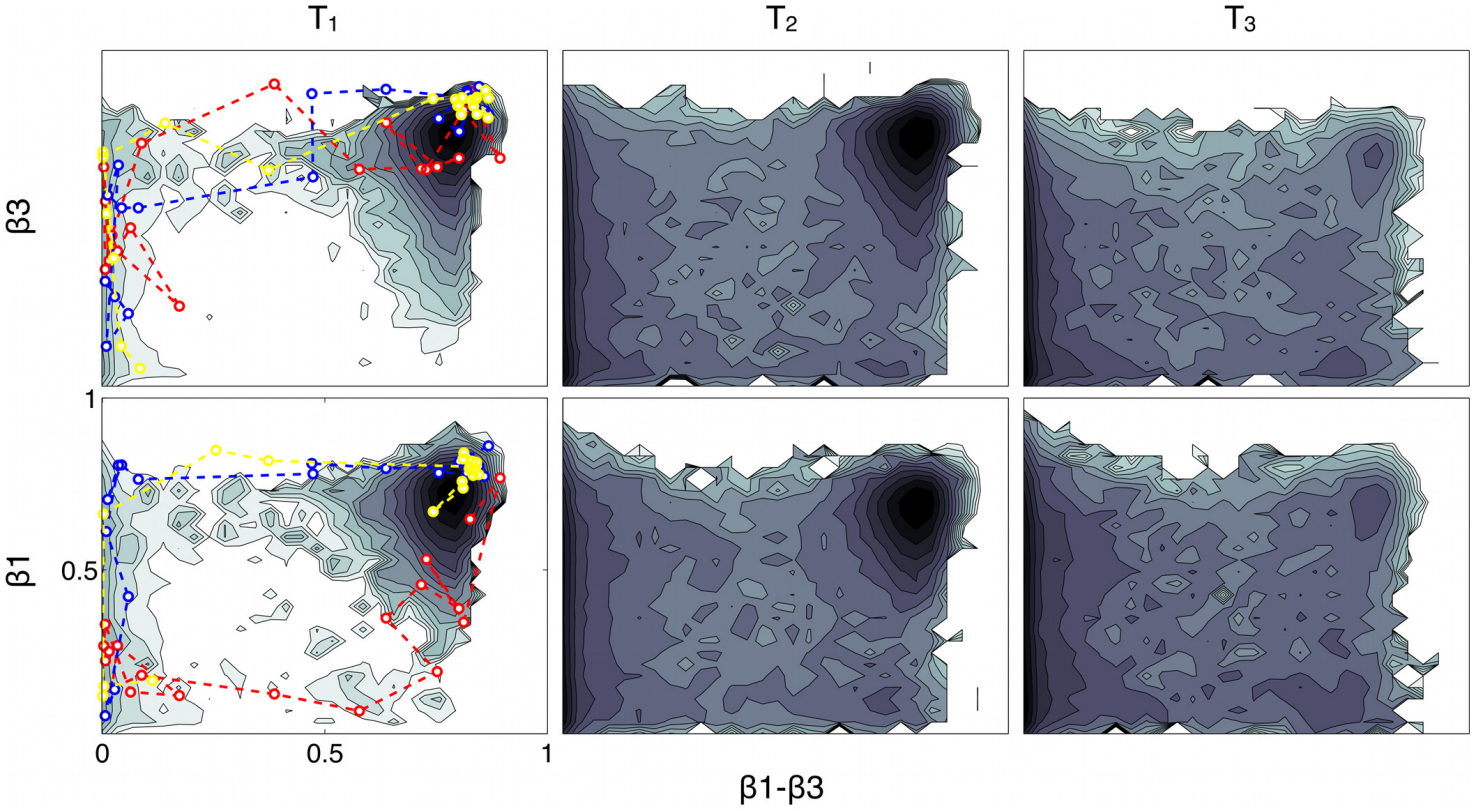
FES as a function of 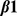
–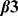 vs. 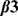 contacts (top panels) and of 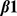
–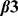 vs. 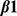 contacts (bottom panels) at T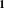 = 0.969, T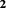 = 0.998 and T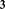 = 1.021. FES are obtained by reweighting the structure-based potential PTMetaD simulation. Isoenergy lines are drawn every 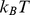. In dashed lines, projection of the relevant part of the all-atom explicit-solvent unfolding simulations at 700K (blue and red) and 500K (yellow).

The remaining steps of the folding process are more complex. The contacts 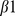
–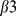 are formed after the hydrophobic collapse (Fig. 4c). When this happens, 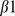 and 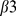 can be either folded or unfolded, depending on T (Fig. 5). At low T, 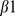 and 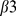 are formed independently and only after they dock forming the contacts 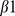
–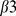. At higher temperatures, the sequence of events can vary and 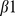
–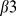 tends to be formed before 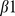 and 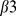 are fully structured.

Lastly, we discuss 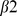 whose behavior is less correlated with the overall folding process. If we examine the 2-D FES involving 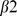, we notice that this part of the protein can be folded independently of the other subunits. For instance, in the FES as function of 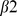 and FN (Fig. 4d) the shallow basins correspond to 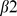 folded whatever FN is formed or not.

## Discussion

The simulations of the HIV–1–PR monomer performed with theoretical models of different complexity suggested that the high-temperature unfolding and the folding mechanism of this protein are heterogenous processes. The remarkable analogies found in the nature and in the fine details of these two processes stimulated us to make mechanistic predictions about the actual folding routes of the monomer. To better visualize these analogies, we have projected the all-atom explicit-solvent unfolding trajectories onto the structure-based potential FES (Fig. 4 and 5).

In our scenario, folding is characterized by a milestone event followed by multiple different pathways leading to the native state. The milestone event corresponds to the assembling of an hydrophobic core rich in Valine, Leucine and Isoleucine residues. This collapse can be represented as a two steps process. First, the FN–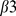 is formed, and then the folding nucleus made by the two LES S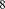 and S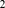 is assembled. This result is suggested both by the simulations with the structure-based potential and by the set of high-temperature unfolding trajectories in explicit solvent. The analysis of the latter clearly shows that the unfolding of the hydrophobic core is the last step before the complete denaturation of the monomer in all pathways. This collapsed structure might be related with the possible presence of an intermediate in the folding process, as proposed in Ref. [3].

Several theoretical works indicate that these fragments play an essential role in the folding process. The hierarchy of contacts formed during a G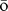-model simulation [12]; a study of cooperative folding units that exhibit a stronger protection against unfolding than other parts of the monomer [47]; a Gaussian network model study of the normal modes around the native conformation [48]; the study of structured fragments in the transition state region detected by 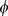-values analysis [5]; the stability temperature associated with each contact of HIV–1–PR [49]; the residues most conserved in those sequences that can successfully fold during a simulation of HIV–1–PR evolution [50].

Further support to these theoretical findings comes from different experimental facts. As common to retrovirus, the mechanism of reproduction of HIV is very fast and at the same time extremely prone to errors. The intrinsic nature of this mechanism together with the pressure induced by drugs led to the appearance of many mutated HIV-1-PR with conserved folding features. An analysis of mutations in 28417 isolates coming from treated and untreated patients infected with HIV showed that only conservative mutations, *i.e.* substitutions with an amino acid with similar chemical properties, affected those residues belonging to the hydrophobic core [51]. Moreover, a study of the degrees of conservation of residues in a family of proteins structurally similar to HIV–1–PR demonstrated that fragments 22–33 and 81–90 were the most conserved regions [52]. These facts support our theoretical findings as evolution is likely to pay a greater attention in conserving those parts of the protein that play a central role in the folding process.

A strong indication that fragment S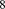 is crucial for folding comes also from the ability of the mimetic peptide p–S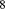 to inhibit the folding of HIV-1-PR. This was demonstrated in standard enzymatic essays that measured the inhibition constant of p-S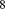 (K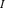 = 2.58
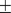
0.78
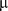
M). Circular dichroism spectroscopy showed that inhibition was accompanied by a strong decrease of the 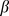-sheet content suggesting that the enzyme was at least partially unfolded [16]. *Ex*–*vivo* experiments with infected cells demonstrated that p–S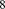 was able to cross the cell membrane, was not toxic to the peripheral blood mononuclear cells and had an antiviral levels below toxic concentration. Consistently with the enzymatic essays, this peptide inhibited the maturation of the virus at a micromolar concentration [17].

The rationale behind the use of p–S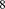 as folding inhibitor is that this peptide may interact with its counterpart S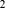 and prevent the formation of FN and eventually the folding of the whole monomer. Our results open the way to another possible interpretation. Instead of interacting with its natural partner S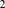, the highly hydrophobic peptide may also disturb the assembling of the entire hydrophobic core. Further experiments on the complex between the protease and the inhibitor could shed light on the details of this process.

After hydrophobic collapse, multiple pathways lead the protein to its native state. Our results suggest that, at physiological condition, the main pathway proceeds through the formation of the two 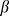-strands 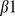 and 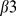 and then their docking into the native conformation. This fact is supported also by our long unbiased simulation. Here the breaking and reforming of the hydrogen bonds pattern between 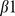 and 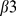 was observed in the time scale of 0.5 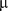
s, while the two subunits remained individually formed. This suggests that the interaction between these two fragments is only marginally stable at room temperature. Moreover, if we examine the main route in the network of high-temperature unfolding pathways (Fig. 3, panel 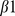
–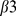), the contacts 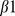
–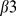 are broken while 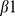 and 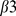 retain their native structure.

The FES obtained from our structure-based potential simulations suggest also that, at temperature lower than 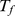, there is no preferential order in which 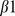 and 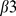 fold (Figure S9). However, 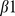 turns out to be the most stable between the two. The stability of 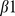 was confirmed by our long unbiased all-atom simulation in explicit solvent and by the sequence of events in the unfolding network. Here this 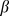-strand remained structured along the main route toward the unfolded state almost until the complete unfolding of the protein. The predominance of this folding route with respect to others is very sensitive to external conditions. As temperature increases, others pathways become more and more populated. In particular the interaction 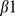
–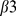 appears to be formed before each subunits folds, and 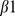 seems to get structured before 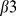.

Our results suggest also that the fragment 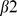 that corresponds to the flap region in the dimer structure retains a behavior almost uncorrelated with the overall folding process. This can be seen in our structure-based potential FES (Fig. 4) and in the network of unfolding pathways (Fig. 3, panel 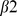). This reflects itself in the flexibility of this fragment in the native state of the monomer (Figure S1). This flexibility is required to accommodate the substrate inside the active site cavity [24]–[28], [53].

Finally, the native state of the monomer appears stable with respect to thermal unfolding. In our 0.5 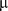
s long unbiased simulation at room temperature the protein retained a very compact structure with root mean square fluctuations on average lower than 1.5 Å, except for the termini and the flap region. These results are compatible with the high unfolding barrier measured experimentally [3].

In conclusion, we have formulated, in a biologically and pharmacologically relevant case, a folding scenario in which multiple pathways and milestones coexist. In this picture, the formation of an hydrophobic core is a milestone event, while the rest of the protein can reach its native state following different pathways and order of assembling. The insight obtained from our simulations, which is supported by several lines of theoretical and experimental evidence, can guide a more rational design of folding-inhibitor drugs as the residues that play a key role in the folding process have been identified. Targeting the formation of the hydrophobic core, being a process common to all the folding pathways, could prove a successful strategy in the fight against AIDS.

## Methods

In the following paragraphs, we provide the details of our all-atom explicit-solvent simulations, of the clustering and network analysis and of the structure-based potential runs. Additional technical information can be found in Text S1.

Throughout all the simulations and analysis, we used a common definition of contact maps. A contact between the 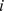
th and 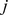
th 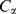 atom of the protein is defined as 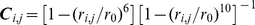 where 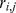 is the distance between the two atoms and 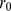 = 8.5 Å [54]. This definition of contact map is different from the one commonly used in literature [54], which is discrete and where 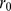 is a sharp cutoff. In order to be used as collective variables in a metadynamics simulation, contacts must be defined in terms of a function with continuos derivatives.

### All-atom simulations

All-atom simulations were carried out using AMBER99SB force field [55] and NAMD 2.7b1 code [56]. The initial configuration was taken from the structure of the HIV–1–PR dimer (PDB code 1BVG). The monomer was solvated in a periodic cubic box of 84 Å using 18957 TIP3P water molecules [57]. The system was pressurized at 1 atm at 300K using a Langevin thermostat and piston for 500 ps. The NVT run was carried out for 512 ns at 300K using a Langevin thermostat. The unfolding analysis was performed on a set of 30 trajectories generated starting from the same equilibrated structure with different initial velocities. The temperature of 700K was enforced using a Langevin thermostat. All the thermal unfolding runs were simulated for 8 ns. The final configurations had a RMSD calculated on the C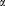 atoms that ranged from 11 to 22 Å from the native structure. An additional simulation at 500K was performed for 1.1 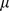
s.

### Clustering and network analysis

The ensemble of configurations produced in the unfolding simulations was clusterized using the k-means algorithm [32], using as distance between two configurations a properly defined distance in the space of contact maps. Two clusters were connected by a link if a transition between them was observed during the unfolding simulations. To visualize the connectivity among clusters, we used Visone [58]. The method used to display the network of clusters was the metric multidimensional scaling [59].

The clustering algorithm used here is based on a choice *a priori* of the number of clusters in which data are organized. We explicitly checked that the sequence of events and the different unfolding pathways found by our analysis were robust with respect to this choice (Figure S4). The final data reported in Fig. 3 were generated using 50 clusters.

### Structure-based potential simulations

Coarse-grained simulations were carried out using the all-atom structure-based potential introduced in Ref. [18] and GROMACS 4 [60] equipped with PLUMED [61]. For the PTMetaD simulation, 16 replicas were distributed with a geometric progression in a temperature range between 0.969 and 1.057 in unit of 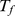 = 113.5K. To keep the target temperature, the stochastic thermostat of Bussi *et al.* [62] was used. Exchanges between configurations were attempted every 200 steps. The total simulation time for each replica was 2

10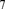 steps. As collective variable, we used the total number of native contacts without any discrimination among our six subsets. Gaussians of 1.0 kjoule/mol height and 5.0 width were deposited every 1000 steps. We monitored the convergence by calculating at different times the free-energy difference between folded and unfolded states (Figure S5). Convergence was accelerated by orders of magnitude with respect to standard PT [22]. To calculate from the biased simulations the multiple FES as a function of the fraction of native contacts of our six descriptors, we used the reweighting algorithm of Ref. [46].

## Supporting Information

Text S1 Additional technical details about the all-atom explicit solvent and coarse-grained simulations, and the reweighting algorithm.
(0.04 MB PDF)

Figure S1 Root mean square fluctuations (RMSF) of Cα atoms during a simulation at room temperature initiated from the crystallographic structure. The RMSF has been calculated using the tool g_rmsf included in the GROMACS 4 package. In the insert, residues with RMSF between 1.0 A and 1.5 A are colored in green while residues with RMSF larger than 1.5 A are colored in red.
(7.06 MB EPS)

Figure S2 Analysis of the all-atom explicit-solvent simulation of HIV-1-PR monomer at room temperature. Top. Time evolution of hydrogen bond numbers between N-terminus (residues 3–7) and C-terminus (residues 95–98). Bottom. Time series of the six sets of native contacts of HIV-1-PR monomer.
(4.56 MB EPS)

Figure S3 Atomistic detail of β1–β3 interaction. During the 0.5 µs NVT simulation starting from the native state, the hydrogen bonds GLU65:H-LYS14:O and GLU65:OE2-LYS14:2HE are broken and reformed.
(4.04 MB EPS)

Figure S4 Network analysis performed with 100 clusters (top) and 200 clusters (bottom). Coloring is done according to the formation of β1.
(3.64 MB EPS)

Figure S5 FES convergence in the PTMetaD run measured as the free-energy difference between folded and unfolded states as a function of time.
(1.94 MB EPS)

Figure S6 FES as a function of the fraction of native contacts of three β-strand subunits of HIV-1-PR at T1 = 0.969, T2 = 0.998 and T3 = 1.021. FES are obtained by reweighting the structure-based potential PTMetaD simulation.
(1.62 MB EPS)

Figure S7 FES as a function of HYDRO and all the other contacts at T1 = 0.969, T2 = 0.998 and T3 = 1.021. FES are obtained by reweighting the structure-based potential PTMetaD simulation. Isoenergy lines are drawn every kBT.
(3.84 MB EPS)

Figure S8 FES as a function of FN and FN-β3 at T1 = 0.969, T2 = 0.998 and T3 = 1.021. FES are obtained by reweighting the structure-based potential PTMetaD simulation. Isoenergy lines are drawn every kBT.
(3.74 MB EPS)

Figure S9 FES as a function of β1 and β3 at T1 = 0.969, T2 = 0.998 and T3 = 1.021. FES are obtained by reweighting the structure-based potential PTMetaD simulation. Isoenergy lines are drawn every kBT.
(3.70 MB EPS)
