# Supplementary material for: Multiple Routes and Milestones in the Folding of HIV–1 Protease Monomer
Source: PLoS One. 2010 Oct 13;5(10):e13208. doi: 10.1371/journal.pone.0013208 (PMC2954147; doi:10.1371/journal.pone.0013208)
Supplement: Text S1 — Additional technical details about the all-atom explicit solvent and coarse-grained simulations, and the reweighting algorithm. (0.04 MB PDF) [file pone.0013208.s001.pdf]

Supporting Information.

Multiple routes and milestones in the folding of HIV–1 protease monomer

Massimiliano Bonomi,[*,†] Alessandro Barducci,[†] Francesco L. Gervasio,[‡] and Michele Parrinello[†]

Computational Science, Department of Chemistry and Applied Biosciences, ETH Zurich, Lugano, Switzerland, and Computational Biophysics Group, Structural Biology and Biocomputing Programme, Spanish National Research Centre, Madrid, Spain

E-mail: mbonomi@ethz.ch

In all our simulations and analysis we used a common definition of contact maps. A contact between the ith and jth C_α atom of the protein is defined as:

$$\boldsymbol{C}_{i,j} = \frac{1-\left(\frac{r_{i,j}}{r_0}\right)^6}{1-\left(\frac{r_{i,j}}{r_0}\right)^{10}}, \tag{1}$$

where $r_{i,j}$ is the distance between the two atoms and r_0 is taken to be $r_0 = 8.5$ Å.[1] This definition of contact map is different from the one commonly used in literature,[1] which is discrete and where r_0 is intended as a sharp cutoff. In order to be used as collective variables in a metadynamics simulation, contacts must be defined in terms of a function with continous derivatives. The definition in Eq. (1) has already been used successfully in studying the folding of small peptides[2,3] and

*To whom correspondence should be addressed
†ETH Zurich
‡Spanish National Research Centre

the interfaces between protein subunits in supramolecular biological assemblies.[4]

All-atom simulations

All-atom simulations were carried out using AMBER99SB force field[5] and NAMD 2.7b1 code.[6] The initial configuration was taken from the structure of the HIV–1–PR dimer (PDB code 1BVG). The monomer was solvated in a periodic cubic box of 84 Å using 18957 TIP3P water molecules.[7] Two chloride ions were added to ensure charge neutrality. A timestep of 2 fs was used. All bonds were constrained using the SHAKE algorithm,[8] with a tolerance of 10^{-8} Å. Electrostatic was treated with the Particle Mesh Ewald method,[9] using a grid spacing of 1 Å, a direct space tolerance of 10^{-6} and an interpolation order equal to 4. The system was pressurized at 1 atm at 300K using a Langevin thermostat with damping coefficient of 5 ps^{-1} and piston for 500 ps.

NVT simulation at room temperature

The NVT run was carried out for 512 ns at 300K using a Langevin thermostat with a damping coefficient of 5 ps^{-1}. The structure of the monomer appeared stable during the whole NVT simulation. The root mean square fluctuations of the C_α atoms were within 0.5 and 1.5 Å along most of the chain (Figure S1). Three regions displayed a grater mobility: the C and N termini and fragment 45–55, corresponding to $\beta 2$. A partial assembling of the N and C terminal into a beta structure was also observed (Figure S2, top panel).

In the bottom panel of Figure S2 we monitor the time series of our six sets of native contacts. In the time scale of the simulation a spontaneous breaking and reforming of the hydrogen-bonds pattern that stabilizes $\beta 1$–$\beta 3$ occurred. Atomistic details of the interaction between $\beta 1$ and $\beta 3$ are reported in Figure S3.

We have calculated the RMSD of the monomer in solution from the structure in the dimer along the first 10 ns of our long NVT run. The RMSD computed on the backbone atoms of all the 99 residues oscillated around 2.2 Å. The RMSD computed only on residues 10 to 90, i.e. excluding

the flexible termini, oscillated around 1.5 Å.

Unfolding analysis

The unfolding analysis was carried out on a set of 30 trajectories generated starting from the same equilibrated structure with different initial velocities. The temperature of 700K was enforced using a Langevin thermostat. All the thermal unfolding runs were simulated for 8 ns. The final configurations had a RMSD from the native structure that ranged from 11 to 22 Å, calculated on the C_α atoms.

To identify common events and main unfolding routes, the ensemble of configurations produced in the unfolding simulations at 700 K was clusterized using the k-means algorithm.[10] This is a simple way to classify a set of N data in a certain number of cluster k fixed *a priori*. The algorithm aims at minimizing the *error squared* function:

$$J = \sum_{j=1}^{k} \sum_{i=1}^{N} \|x_i^{(j)} - c_j\|^2, \tag{2}$$

where $\|x_i^{(j)} - c_j\|^2$ is the distance between a data point $x_i^{(j)}$ and the cluster centre c_j. Here we chose as distance between two configurations x_1 and x_2 the distance in the space of contact maps:

$$\|x_2 - x_2\|^2 = \sum_{j=1}^{6} \frac{1}{N_j} \sum_{i=1}^{N_j} (C_i^j(1) - C_i^j(2))^2, \tag{3}$$

where the index j runs over the six sets of contacts in which we classified the native map, C_i^j is the ith element of this set, as defined in Eq. (1), N_j the total number of contacts belonging to this group.

Two clusters were connected by a link if a transition between them was observed during the unfolding simulations. To visualize the connectivity among the clusters found, we used Visone.[11] The method used to display and organize the network of clusters was the metric multidimensional scaling. The clustering algorithm used here is based on a choice *a priori* of the number of clusters

in which data are organized. We explicitly checked that the sequence of events and the different unfolding pathways found by our analysis were robust with respect to this choice (Figure S4).

Structure-based potential simulations

Coarse grained simulations of the HIV–1–PR monomer were carried out using the all-atom structure-based potential introduced in Ref.[12] and GROMACS 4[13] together with PLUMED.[14] To build the topology, the web server of Onuchic research group (http://sbm.ucsd.edu/) was used.

A time step of 0.0005 in reduced unit and the stochastic thermostat of Bussi *et al.*[15] were used. A thermostat that yields the correct energy fluctuations of the canonical ensemble is crucial in parallel tempering simulations.[16] For the PTMetaD simulation, 16 replicas were distributed with a geometric progression in a temperature range between 0.969 and 1.057 in unit of $T_f = 113.5K$. Exchanges between configurations were attempted every 200 steps. The total simulation time for each replica was $2 \cdot 10^7$ steps. As collective variable, we used the total number of native contacts Q without any discrimination among our six subsets. The ratio between the fictitious temperature of the collective variable $T + \Delta T$[17] and that of the simulation T was kept constant across the different replicas:

$$\gamma = \frac{T + \Delta T}{T} = 15. \tag{4}$$

Gaussians of 1.0 kjoule/mol height and 5.0 width were deposited every 1000 steps. We monitored the convergence of the PTMetaD simulation by calculating at different times the free-energy difference between folded and unfolded states (Figure S5):

$$\Delta F(t) = -\frac{1}{\beta} \ln \left(\frac{\int_{0.5}^{1} e^{\beta \frac{\gamma}{\gamma-1} V(Q,t)}\, dQ}{\int_{0}^{0.5} e^{\beta \frac{\gamma}{\gamma-1} V(Q,t)}\, dQ} \right) \tag{5}$$

where $\beta = (k_B T)^{-1}$ and $V(Q,t)$ is the metadynamics bias potential acting at time t on the collective variable Q. The definition of folded ($Q \geq 0.5$) and unfolded ($Q < 0.5$) is arbitrary.

Convergence was accelerated by orders of magnitude with respect to standard PT.[18]

Reweighting procedure

To calculate from the PTMetaD runs the multiple FES as a function of the fraction of native contacts of our six descriptors, we used the reweighting algorithm introduced in Ref.[19] This method is based on the evolution of the biased probability distribution $P(\boldsymbol{R},t)$ during the metadynamics simulation:

$$P(\boldsymbol{R},t+\Delta t) = e^{-\beta(\dot{V}(\boldsymbol{s}(\boldsymbol{R}),t)-\langle\dot{V}(\boldsymbol{s},t)\rangle)\Delta t} P(\boldsymbol{R},t), \tag{6}$$

where $\dot{V}(\boldsymbol{s}(\boldsymbol{R}),t)$ is the time derivative of the bias potential and the average in the exponent is calculated in the biased ensemble. In the long time limit, the Boltzmann distribution $P_B(\boldsymbol{R})$ can be recovered using a standard umbrella sampling reweighting:

$$P_B(\boldsymbol{R}) \propto e^{\beta V(\boldsymbol{s}(\boldsymbol{R}),t)} \cdot P(\boldsymbol{R},t). \tag{7}$$

References

(1) Vendruscolo, M.; Najmanovich, R.; Domany, E. *Phys. Rev. Lett.* **1999**, *82*, 656–659.

(2) Bonomi, M.; Branduardi, D.; Gervasio, F. L.; Parrinello, M. *J. Am. Chem. Soc.* **2008**, *130*, 13938–13944.

(3) Bonomi, M.; Gervasio, F. L.; Tiana, G.; Provasi, D.; Broglia, R. A.; Parrinello, M. *Biophys. J.* **2007**, *93*, 2813–21.

(4) Pfaendtner, J.; Voth, G. A. *Biophys. J.* **2008**, *95*, 5324–5333.

(5) Hornak, V.; Abel, R.; Okur, A.; Strockbine, B.; Roitberg, A.; Simmerling, C. *Proteins* **2006**, *65*, 712–725.

(6) Phillips, J. C.; Braun, R.; Wang, W.; Gumbart, J.; Tajkhorshid, E.; Villa, E.; Chipot, C.; Skeel, R. D.; Kalé, L.; Schulten, K. *J. Comput. Chem.* **2005**, *26*, 1781–802.

(7) Jorgensen, W. L.; Chandrasekhar, J.; Madura, J. D.; Impey, R. W.; Klein, M. L. *J. Chem. Phys.* **1983**, *79*, 926–935.

(8) Ryckaert, J. P.; Ciccotti, G.; Berendsen, H. J. C. *J. Comput. Phys.* **1977**, *23*, 327–341.

(9) Essmann, U.; Perera, L.; Berkowitz, M. L.; Darden, T.; Lee, H.; Pedersen, L. G. *J. Chem. Phys.* **1995**, *103*, 8577–8593.

(10) Lloyd, S. P. *Ieee T. Inform. Theory* **1982**, *28*, 129–137.

(11) Brandes, U.; Wagner, D. Visone: Analysis and Visualization of Social Networks. In *Graph Drawing Software*; Jünger, M., Mutzel, P., Eds.; Springer: Berlin, Germany, 2003; pp 321–340.

(12) Whitford, P. C.; Noel, J. K.; Gosavi, S.; Schug, A.; Sanbonmatsu, K. Y.; Onuchic, J. *Proteins* **2008**, *75*, 430–441.

(13) Hess, B.; Kutzner, C.; van der Spoel, D.; Lindahl, E. *J. Chem. Theory Comput.* **2008**, *4*, 435–447.

(14) Bonomi, M.; Branduardi, D.; Bussi, G.; Camilloni, C.; Provasi, D.; Raiteri, P.; Donadio, D.; Marinelli, F.; Pietrucci, F.; Broglia, R. A.; Parrinello, M. *Comp. Phys. Comm.* **2009**, *180*, 1961–1972.

(15) Bussi, G.; Donadio, D.; Parrinello, M. *J. Chem. Phys.* **2007**, *126*, 014101.

(16) Rosta, E.; Buchete, N.; Hummer, G. *J. Chem. Theory Comput.* **2009**, *5*, 1393–1399.

(17) Barducci, A.; Bussi, G.; Parrinello, M. *Phys. Rev. Lett.* **2008**, *100*, 020603.

(18) Bonomi, M.; Parrinello, M. *Phys. Rev. Lett.* **2010**, *104*, 190601.

(19) Bonomi, M.; Barducci, A.; Parrinello, M. *J. Comput. Chem.* **2009**, *30*, 1615–1621.
